# 1548. Evaluation of rural HIV screening in adults with one or more risk factors

**DOI:** 10.1093/ofid/ofad500.1383

**Published:** 2023-11-27

**Authors:** Finlay Pilcher, Eleanor Stedman, Bradley Tompkins, Andrew Hale, Devika Singh

**Affiliations:** Larner College of Medicine at the University of Vermont, Burlington, Vermont; University of Vermont Medical Center, Burlington, Vermont; University of Vermont Medical Center, Burlington, Vermont; University of Vermont, Burlington, Vermont; The University of Vermont Medical Center, Burlington, Vermont

## Abstract

**Background:**

The National HIV/AIDS Strategy aims to end the HIV epidemic in the United States by 2030. However, despite significant medical advances in HIV prevention (specifically treatment as prevention), transmission without knowledge of HIV status remains a significant barrier to reducing new infections. Developing effective and equitable HIV screening methods is an essential step towards HIV elimination.

Individuals residing in rural settings across the U.S. have limited access to HIV-literate providers due to complex and multifactorial barriers including long travel distances to appointments, fewer community support resources, and scarcity of specialized HIV care providers. The opt-out approach to HIV screening may be insufficient to adequately reach people living in rural communities, particularly those with elevated risk of HIV transmission.

**Methods:**

Our study used Vermont-specific data from the Centers for Disease Control and Prevention Behavioral Risk Factor Surveillance System (BRFSS) surveys from 2010 to 2012 and 2016 to 2019. We included participants who responded to three questions in the HIV/AIDS section of the BRFSS. BRFSS surveys from 2013 to 2015 omitted one of the three questions and therefore were excluded from the study.

Participants were assigned either a low- or high-risk category based on a question which asked whether they engaged in certain behaviors associated with HIV transmission (e.g., intravenous drug use, condomless anal sex, etc.) over the past year. HIV screening was considered adequate for participants in the low-risk category if they had one or more lifetime HIV test. Participants in the high-risk category were deemed adequately screened if they had been tested for HIV in the past year.

**Results:**

The results of the analysis are shown in Table 1. Pearson’s chi-squared value (χ2) was 5.1 with an associated p-value = 0.024 suggesting that the percentage of adequate screening varies significantly with HIV risk category.

**Figure:**
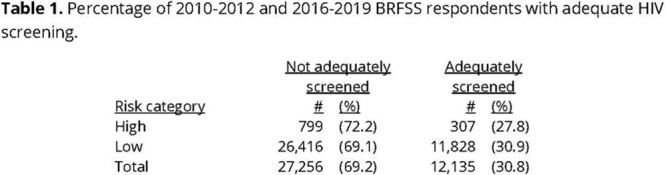

**Conclusion:**

Our results suggest that HIV screening remains suboptimal in Vermont, particularly for those at increased risk of transmission. These findings indicate that more inclusive and expansive strategies including targeted HIV screening may be warranted in rural regions.

**Disclosures:**

**All Authors**: No reported disclosures

